# Micro-scale interactions between Arabidopsis root hairs and soil particles influence soil erosion

**DOI:** 10.1038/s42003-020-0886-4

**Published:** 2020-04-03

**Authors:** Sarah De Baets, Thomas D. G. Denbigh, Kevin M. Smyth, Bethany M. Eldridge, Laura Weldon, Benjamin Higgins, Antoni Matyjaszkiewicz, Jeroen Meersmans, Emily R. Larson, Isaac V. Chenchiah, Tanniemola B. Liverpool, Timothy A. Quine, Claire S. Grierson

**Affiliations:** 1 KU Leuven, Oude Markt 13-bus 5005, 3000 Leuven, Belgium; 2 School of Biological Sciences, Life Sciences Building, University of Bristol, 24 Tyndall Avenue, BS8 1TQ Bristol, UK; 3 School of Mathematics, University of Bristol, Fry Building, Bristol, BS8 1UG UK; 4 School of Engineering Mathematics, Queen’s Building, University of Bristol, Bristol, BS8 1TR UK; 5 TERRA Teaching and Research Centre, Gembloux Agro-Bio Tech, University of Liège, Gembloux, 5030 Belgium; 6 Geography, College of Life and Environmental Sciences, University of Exeter, Amory Building, Rennes Drive, Exeter, EX4 4RJ UK

**Keywords:** Plant physiology, Biophysics

## Abstract

Soil is essential for sustaining life on land. Plant roots play a crucial role in stabilising soil and minimising erosion, although these mechanisms are still not completely understood. Consequently, identifying and breeding for plant traits to enhance erosion resistance is challenging. Root hair mutants in *Arabidopsis thaliana* were studied using three different quantitative methods to isolate their effect on root-soil cohesion. We present compelling evidence that micro-scale interactions of root hairs with surrounding soil increase soil cohesion and reduce erosion. Arabidopsis seedlings with root hairs were more difficult to detach from soil, compost and sterile gel media than those with hairless roots, and it was 10-times harder to erode soil from roots with than without hairs. We also developed a model that can consistently predict the impact root hairs make to soil erosion resistance. Our study thus provides new insight into the mechanisms by which roots maintain soil stability.

## Abstract (web summary)

Sarah De Baets et al. investigate the effect of root hair abundance on root-substrate cohesion and experimental soil erosion in Arabidopsis. They find that plants with root hairs are more difficult to detach from soil and are better able to prevent soil erosion than root hairless plants and present a model to predict the impact of root hairs on soil erosion resistance.

## Introduction

Soil erosion rates associated with agricultural intensification and expansion are 100–1000 times higher than natural background erosion and much higher than soil formation, posing a serious threat to sustainable agriculture, food and environmental security^1,2^. Soil erosion is likely to worsen as the global population grows, average calorific intake increases and climate changes. Plants limit erosion by sheltering soil from erosive forces with their aerial parts and binding soils with their roots, both of which help to retain soil on slopes and anchor plants in the ground^3–5^. Plant root system architecture develops in response to local nutrient concentrations and precision nutrient placement has been mooted as a means of controlling soil erosion^6^. The selection of cultivars best suited to resisting erosion could be part of future sustainable soil management; however, this requires the identification of erosion-resistant traits. While the importance of meso-scale root properties of plant species (e.g. length, diameter, surface area and tensile strength) that support soil erosion resistance has been well studied experimentally and through modelling^3,4,7,8^ the understanding of the potential role of root micro-scale properties (e.g. root hairs, which are typically up to 1 mm long and tens of microns across) in controlling emergent soil properties like soil erosion resistance is limited.

There is a growing awareness that micro-scale processes at the root–soil interface (rhizosphere) are important for determining the properties and functions of soils and ecosystems that support sustainable agricultural land use and management. Symbiosis between roots and mycorrhizal fungi, for example, positively affect water balance, energy balance, nutrient/element cycling and soil hydrophobicity^9,10^. Likewise, root hairs have been linked to phosphate uptake, rhizosphere soil structure formation^11^, root penetration^12^, water uptake^13^ and rhizosheath (i.e. the weight of soil adhering strongly to roots upon excavation) formation in crop plants^14^. Plant roots also secrete compounds (exudates) that have been shown to promote soil aggregation^15^, supporting a composite-like medium consisting of soil particles, plant roots, and plant- and microbe-derived compounds that all contribute to mutual cohesive interactions. Nevertheless, there is no current convincing evidence for micro-scale root properties such as root hairs in soil cohesion. Indeed, the presence of root hairs is required for rhizosheath formation, but the effect of root hair length on rhizosheath strength and size has not been detected^14^.

Previous studies have explored the mechanisms of adhesion of roots to soil and cohesion of the root-soil composite by comparing species with different root architecture to evaluate how thick, deep roots; thin roots; and dense, fine roots change soil erosion resistance^3–5^. The technical term ‘cohesion’ refers to the tendency of the ‘root–soil matrix’ (which is a composite material of soil particles, plant roots, plant-derived compounds and microbes) to maintain mechanical integrity^16^. Thus, root–soil cohesion includes both roots adhering to soil as well as soil particles sticking to one another as an effect of root exudation or plant root–microbe interactions.

The explanatory power of prior studies is limited because root–soil cohesion may be influenced by inter-species differences other than those selected, especially differences in root micro-architecture. We overcome these limitations by using mutants and transgenic lines of the model plant *Arabidopsis thaliana* and novel root–gel attachment and uprooting resistance assays, as well as an established soil erosion assay in conjunction with a mathematical model to quantify the soil cohesion effects of root hairs. Our work advances the quantitative understanding of how root hairs affect root–soil cohesion and have a measurable effect on soil erosion resistance.

## Results

**Fig. 1:**
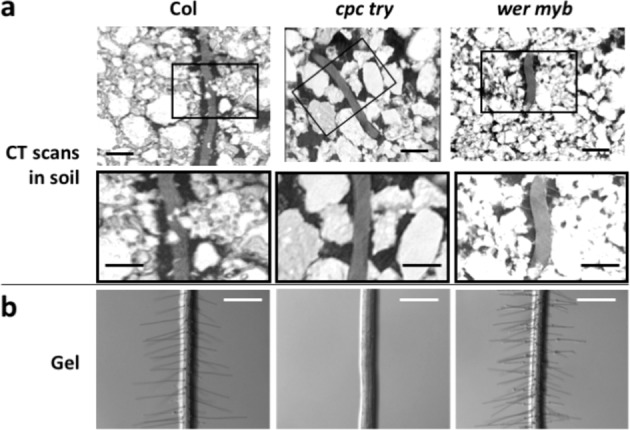
Root hair phenotypes that affect plant–soil cohesion in Arabidopsis. Root hair phenotypes of wild type, *cpc try* and *wer myb23 Arabidopsis thaliana* grown in **a** a clay–loam soil or on **b** gel medium. Black boxes in the upper panels in **a** indicate the regions magnified in the lower panels. Images were produced as described in the Methods using **a** a Nikon XT H 225 ST CT scanner (settings: energy: 90 kV, current 60 (μA) exposure 1 s, 5 frames averaged per projection, voxel size = 0.00278056) and **b** bright field, high contrast lighting on a Leica MZ FLIII microscope. Scale bar = 1 mm.

To characterise the role of root hairs in plant–substrate cohesion, our assays included the use of Arabidopsis wild type (Col-0) and root hairless or root hair overproducing mutant lines^17–19^. Transgenic *35S::RSL4* plants have longer root hairs^18^ and *wer myb23* seedlings produce more root hairs than wild-type seedlings^20^, while the *rsl4-1* mutant seedlings have a decreased number of short roots hairs^18^ and *cpc try* mutant seedlings do not produce root hairs^21^. In soil, the *cpc try* roots had 97% less dense network of root hairs compared to wild type, whereas the root hair density was 1.6 times higher for *wer myb23* compared to wild type (Table 1, Fig. 1). We confirmed that the root architecture of 10- to 11-day-old wild type, *cpc try* and *wer myb23* plants had no observable difference in lateral root length, lateral root count, rooting depth and vertical angle from the root system, which indicates that the only significant difference between these lines is root hair growth (Table 1, Fig. 1).

**Table 1:** Main root hair phenotypic differences between wild type (Col-0), *cpc try* and *wer myb* grown in gel or clay soil.

Substrate	Mutant line	Root hair density (cm^−1^)	Root hair length (mm)	Depth of system (cm)
Gel	*cpc try*	Hairless in all observations, *N* = 90	—	9% deeper than wild type *P* = 0.002 *N* = 35 Mean (±1 SD) of *cpc try* = 10.39 (±1.41) Mean (±1 SD) of wild type = 9.57 (±1.3)
	*wer myb*	84% denser *P* < 0.001 *N* = 90 Mean (±1 SD) of *wer myb* = 304 (±45) Mean(±1 SD) of wild type = 165 (±22)	36% longer *P* = 0.019 *N* = 30 Mean (±1 SD) of *wer myb* = 1.75 (±0.88) Mean(±1 SD) of wild type = 1.29 (±0.56)	—
Clay soil	*cpc try*	97% less dense *P* < 0.001 *N* = 9 Mean (±1 SD) of *cpc try* = 2.22 (±6.66) Mean (±1 SD) of wild type = 63.64 (±8.09)	N/A	N/A
	*wer myb*	62% denser *P* < 0.001 *N* = 10 Mean (±1 SD) of *wer myb* = 103.33 (±20.58) Mean (±1 SD) of wild type = 63.64 (±8.09)	N/A	N/A

All results in the table are comparisons to wild type using ANOVA. Dashes indicate no significant difference and N/A indicates data could not be collected. Lateral root length, lateral root count and vertical angle from the root system were also measured on gel and had no significant difference from wild type. Root hairs on gel were images under magnification with dark field lighting. Photographs of whole root systems in gel were analysed with a combination of ImageJ^46^ and Root Nav^61^. Root hair counts in soil were performed on 1-mm-long root sections from CT images such as those in Fig. 1.

### Root hairs contribute to root–substrate cohesion

**Fig. 2:**
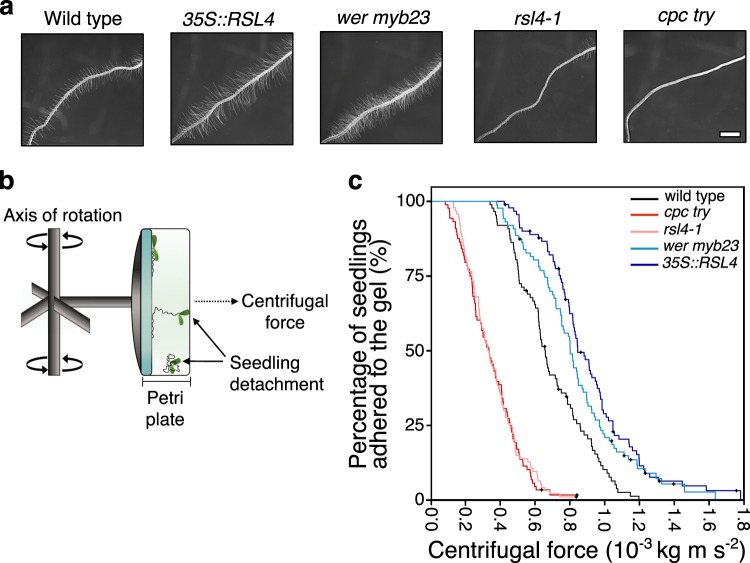
Root hairs increase root adherence to a gel substrate. **a** Roots of 5-day-old wild type, long haired *35S::RSL4*, root hair overproducing *wer myb23*, sporadic and short haired *rsl4-1*, and hairless *cpc try* seedlings. Scale bar =1 mm. **b** A schematic of the Arabidopsis centrifugation root–gel adhesion assay to illustrate the centrifuge rotor and swinging bucket containing an inverted Petri plate. Ten seedlings/plate were grown on the surface of the gel medium and as the centrifuge rotates, the bucket swings out so that the plate is perpendicular to the rotor. Over a period of ~10 min, the plates are exposed to 1-min pulses of increasing centrifugal forces and the proportion of detached seedlings are scored between each speed setting. Illustration not to scale. **c** Survival curves showing the proportion of seedlings that remained adhered to the gel at increasing centrifugal force for 87 wild type (black); 88 *cpc try* (red); 94 *rsl4-1* (pink); 87 *wer myb23* (light blue); and 91 *35S::RSL4* (dark blue) 5-day-old seedlings. The angular velocity (*ω*) and diameter of the centrifuge, together with the aerial tissue weight of each seedling are used to calculate the centrifugal force (mass × radius × *ω*2 = Fc, kg m s^−2^) resisted by each seedling. The seedlings with more and longer root hairs were able to remain attached to the medium over the course of the experiment compared to wild type, while the seedlings with fewer or no root hairs did not. Black crosses represent plants that remained adhered to the gel medium after the maximum centrifugal speed (1611 RPM). Results are from one representative experiment of at least two independent batches, which each included over 70 biological replicates for each genotype.

We developed a centrifugal assay that measures the strength of root–gel adhesion in Arabidopsis seedlings with and without root hairs (Fig. 2). Seedlings were grown vertically on the surface of a sterile, solidified growth medium in Petri plates. After 5 days of growth, the mutant phenotypes were visualised (Fig. 2a) and seedlings were subject to incremental increases in centrifugal force to determine the proportion of seedlings that peeled away from the gel surface between each force interval. Even at the slowest rotation, seedlings experienced a centrifugal force at least 40 times gravity and because the aerial tissue mass of each seedling contributes to the force the roots experience, we incorporated aerial tissue mass when calculating the force (Fc) applied to each seedling (Eq. (2) in Methods; Fig. 2b). To compare the attachment of root hair-defective and root hair-overproducing lines relative to wild-type plants, we used a Cox hazard function regression model^22^ and report the *P* value of the Wald statistic (*z*), the hazard ratio and the lower and upper bound confidence intervals of the hazard ratio. Using this assay, we observed that the *35S::RSL4* and *wer myb23* lines were more resistant to detachment from the gel medium than wild-type plants (Fig. 2c), with a risk of detachment that was 0.44 and 0.56 times that of the control, respectively (*35S::RSL4* – *z* = −5.029, *P* < 0.001, HR = 0.444, 95% CI = 0.324–0.610; *wer myb23* –* z* = −3.705, *P* < 0.001, HR = 0.553, 95% CI = 0.404–0.757). Conversely, the risk of detachment for *rsl4-1* and *cpc try* mutants was 5 and 5.4 times more relative to wild-type plants (*rsl4-1* –* z* = 10.732, *P* < 0.001, HR = 6.002, 95% CI = 4.327–8.325; *cpc try z* = 10.823, *P* < 0.001, HR = 6.369, 95% CI = 4.554–8.906), respectively (Fig. 2c). These results indicate that Arabidopsis seedlings with root hairs (wild type, *35S::RSL4, wer myb23*) are more difficult to detach from sterile gel than seedlings that have no (*cpc try*) or a decreased root hair number and length (*rsl4-1*). Therefore, root hairs directly contribute to plant adhesion during plant growth on solid gel medium.

### Root hairs contribute to plant anchoring in soil

**Fig. 3:**
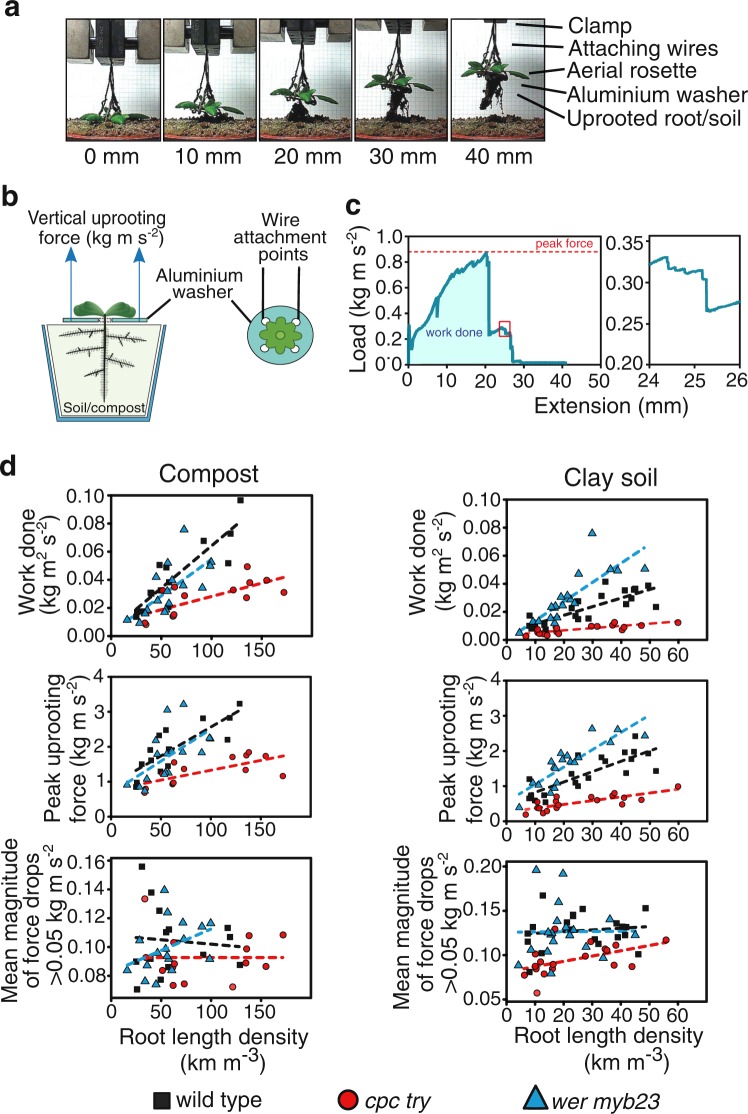
Root hairs increase uprooting resistance from compost and soil. **a** A wild-type Arabidopsis seedling being uprooted by a tensile testing machine illustrates how the cables are anchored to a washer that the mature plant has grown through. As the tensile machine uproots the plant by retracting the cables attached to the washer, the force required to remove the plant from the soil is recorded. **b** Schematic diagram showing a soil-grown Arabidopsis plant grown through an aluminium washer for tensile machine wire attachment. The rosette of the mature plant stabilised the washer so that the force required to uproot the plant could be measured. **c** Representative plot of load (kg m s^−2^) against displacement during the uprooting of the Arabidopsis plant from a clay soil shown in **a**. The adjacent panel shows the portion of the trace enclosed by the red box. **d** Plots of the total work done (i.e. area under curve in **c**), peak force and magnitude of force drops during the uprooting of wild type (black squares), hairless *cpc try* (red circles) and hairy *wer myb23* (blue triangles) mutant plants from compost and a clay soil. Thirteen wild-type plants, 16 *wer myb23* plants and 13 *cpc try* plants were grown in compost, and 17 plants of each genotype were grown in clay soil. In both soil conditions, the presence of root hairs increased the amount of force needed to uproot plants compared to when root hairs were absent.

We performed uprooting assays to investigate whether Arabidopsis root hairs contribute to root–soil cohesion. Plants from each genotype were grown in soil for 3–4 weeks and then uprooted from either a compost–sand mixture or clay soil using a tensile testing machine to record uprooting resistance of the different genotypes (Fig. 3a–c). After uprooting, the plant material was recovered and the root length density (RLD, km m^−3^) of each plant was calculated. Since the root–soil system responds to the uprooting force by a combination of deformation and damage, the maximum force and total energy expended to dislodge the plant from its substrate are macroscopic measures of the strength of root–soil cohesion (Fig. 3c). We identified differences in the total energy in Joules (kg m^2^ s^−2^ m^−1^ root) and maximum pulling resistance (kg m s^−2^ m^−1^ root) required to uproot wild type and mutant plants (Supplementary Table 1).

The root hair overproducing *wer myb23* plants grown in clay soil required a greater maximum uprooting force per RLD and greater total energy per RLD for uprooting compared to wild-type plants (*t* = 2.605, *P* < 0.05 and *t* = 3.807, *P* < 0.001, respectively; Fig. 3d). However, when wild type and *wer myb23* were grown in compost, no detectable difference in uprooting force was observed (Table S2). In contrast, the hairless *cpc try* plants had a lower maximum uprooting force and required total energy to uproot them from clay soil (*t* = −3.034, *P* < 0.05 and *t* = −2.814, *P* < 0.001, respectively) and compost (maximum uprooting force, *t* = −2.394, *P* < 0.05; total energy, *t* = −3.618, *P* < 0.001, respectively) compared to wild-type plants (Fig. 3d). The magnitudes of incremental vertical drops in force during uprooting were measured and indicated that smaller force drops occurred when root hairless *cpc try* plants were uprooted from clay soil (*t* = −4.300, *P* < 0.001) compared to wild type (Fig. 3d). No difference in the uprooting force was observed between *wer myb23* and wild-type plants grown in compost, indicating that root hairs provide resistance in soil until root tissues snap, which is reflected by higher force drops. While root hair overproduction in *wer myb23* increased root–soil cohesion in clay soil compared to wild type, it had no detectable benefit in compost (Fig. 3d), suggesting that composition and structure of the anchoring medium can affect root–soil cohesion behaviour.

### Root hairs affect soil erosion rates

**Fig. 4:**
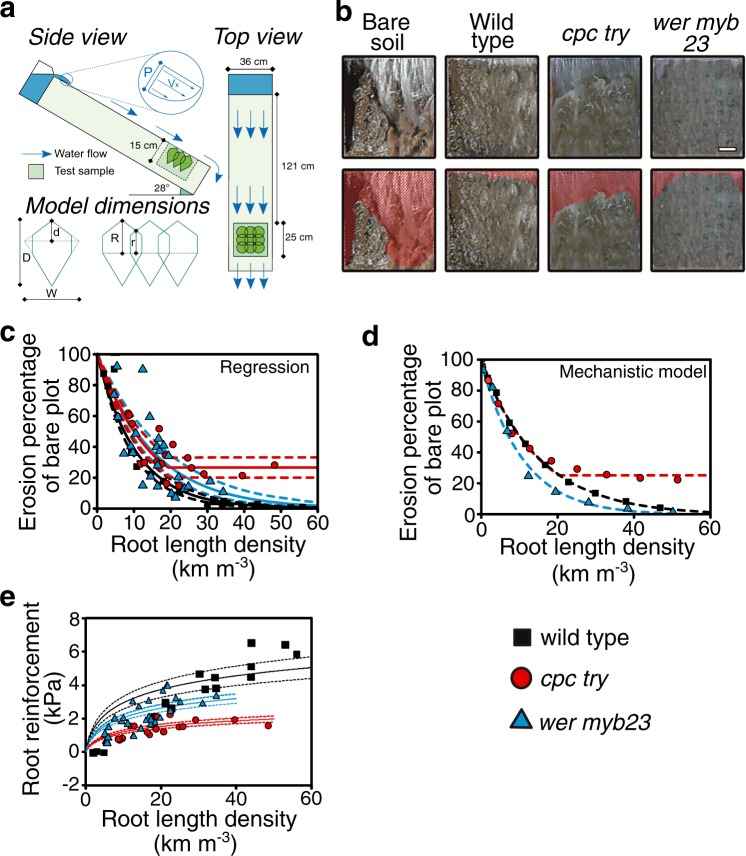
Root hairs reduce soil erosion. **a** Schematic diagram of water flume and soil box used to test the erodibility of bare soil and soil through which roots of wild type, hairless *cpc try* and hair overproducing *wer myb23* plants were grown. Green shapes indicate positions of plants at a density of nine plants per soil box; the green shaded box shows in 2D the space occupied by soil containing Arabidopsis roots. Model dimensions define the root system shape parameters used in the mechanistic model (see Methods for parameter definitions). **b** Representative images of bare soil and soil–root blocks containing 22, 22 and 21 km m^−3^ of wild type, *cpc try* and *wer myb23* roots, respectively (stills taken from Supplementary Movie 1). Scale bar = 5 cm. Upper panels show how approximately 150 L of water flown over these blocks eroded sections of soil, highlighted by red shading in lower panels. **c** Empirical model describing erosion reduction as a function of root length density (RLD) for wild type (black), *cpc try* (red) and *wer myb23* (blue) mutants grown in clay–loam soil. See Results for exponents of the empirical regression lines and goodness of fit for each plant genotype. For RLD > 19, *cpc try* data were modelled using a linear regression with constant term 0.268 ± 0.033. Dashed lines represent the 95% model error bounds computed by Monte Carlo simulation. Markers represent measured erosion reduction rates and corresponding root length densities (RLD, km m^−3^). **d** Output of mechanistic model illustrates either exponential or exponential crossing over to linear dependence of erosion reduction as a function of RLD for plant type. **e** Modelled root reinforcement (kPa) of clay–loam root-reinforced soils as a function of root length density (RLD, km m^−3^) for wild type, *cpc try* and *wer myb23* plants. Regression models, represented as lines, for root reinforcement are 1.23 × LN(RLD + 1) (*R*^2^ = 0.70), 0.50 × LN(RLD + 1) (*R*^2^ = 0.40) and 0.86 × LN(RLD + 1) (*R*^2^ = 0.51), for wild type, *cpc try* and *wer myb23*, respectively. Dashed lines represent 95% model error bounds (Monte Carlo simulation). For **c**, **d** and **e**, *n* = 18 soil boxes containing wild-type roots, 17 (*cpc try*) and 27 (*wer myb23*).

We tested whether root hairs contribute to soil water erosion resistance by comparing the erosion rates of clay–loam soil sown with wild type, hairless *cpc try* or hair overproducing *wer myb23* plants. Plants were grown in 250 × 250 × 150-mm soil boxes over a range of densities (144–1600 m^−2^) for 4–6 weeks. After removing the aerial plant tissue, 150 L of water were flowed over the soil–root blocks for a maximum of 110 s to simulate an overland flow event (Fig. 4a). RLD ranged between 3–56, 8–48 and 5–34 km m^−3^ for wild type, *cpc try* and *wer myb23*, respectively, which correspond with topsoil RLD ranges (1–45 km m^−3^) of six common cover crop species measured in field conditions^23^. We observed that soil–root blocks that contained root length densities >19 km m^−3^ of either wild type or *wer myb23* roots reduced erosion rates to almost zero or 0.27 times that of the bare soil controls, respectively (Fig. 4b; Supplementary Movie 1). For root length densities <19 km m^−3^ that corresponded to <850 plants m^−2^ planting densities, soil erosion decreased exponentially with increased RLD for all mutants (Fig. 4c). The exponents of the empirical regression lines and goodness of fit for wild type, *cpc try* and *wer myb23* were −0.095 ± 0.007 (R2 = 0.96), −0.069 ± 0.007 (R2 = 0.57) and −0.066 ± 0.008 (R2 = 0.62), respectively. At RLD > 19 km m^−3^ that corresponded with plant densities >850 plants m^−2^, hairless *cpc try* was best modelled using a linear regression with the constant term 0.268 ± 0.033, which indicated that there was no further erosion reduction with RLD > 19 km m^−3^ for plants without root hairs (Fig. 4c). In contrast, plots planted with *wer* myb23 lines reduced soil erosion to 0.19, 0.10 and 0.05 times that of bare soil rates at 25, 35 and 45 km m^−3^, respectively (Fig. 4c, d). Erosion rates in wild-type plots were reduced to 0.09, 0.04 and 0.01 times that of the bare soil rates at 25, 35 and 45 km m^−3^, respectively (Fig. 4c, d). These results show that at high root densities, roots with root hairs reduced erosion rates to near zero, while erosion rates of soils containing hairless roots were only reduced to 0.25 times that of bare soil.

The regression models fitted through the experimental erosion data were robust with relatively narrow 95% error bounds (simulated with Monte Carlo), especially for wild-type plants (Fig. 4c). Despite evidence that hairless roots provide limited erosion resistance, the overproduction of hairs by *wer myb23* does not offer an erosion resistance advantage over wild-type root hair production. Wild-type roots confer erosion resistance at the upper end of the range observed and with lower variance.

Erosion rates from a modelled intensive summer rainstorm (peak rainfall intensity of 60 mm h^−1^) indicated that a 3 kPa soil cohesion increase due to the presence of *Lolium perenne* roots could reduce soil loss to 0.015 that of bare soil^7^. Therefore, we determined soil reinforcement values in the presence of roots with different root hair densities/phenotypes using the same method employed previously^7^ for each soil–root block (Fig. 4e). Using regression parameters, we calculated that wild type and *wer myb23* roots increased soil cohesion by approximately 3.7 and 2.6 kPa at 19 km m^−3^ RLD, respectively, whereas soil reinforced with hairless roots at a similar RLD only improved soil cohesion by 1.5 kPa (Fig. 4e). The modelling and experimental observations suggest that the soil reinforcement effect of root hairs has a quantitative impact on soil cohesion values that serve as input for erosion models and, hence, have relevance to soil erosion predictions at field and landscape scales.

### Mechanistic model describing erosion response to root hairs

We developed a mechanistic model that simulates erosion response to changes in RLD and root hair expression (Fig. 4d). This model enabled us to explore previously unaddressed variations in soil cohesion at the scale of roots and root hairs. In the model, we used information of root system architecture^24,25^ to account for the heterogeneous cohesion of root-reinforced soil, where resistance to detachment is strongest along the primary root, decreases radially outwards, and depends on rooting depth (Fig. 4a). The effectiveness of the root hairs to enhance soil cohesion is represented by the function *γ*, described by parameters *M*_1_ and *M*_2_ (Eq. (11) in Methods), where the maximum root hair enhancement (*M*_max_) is represented by *M*_1_/*M*_2_. Using the model to simulate our experimental observations, we deduced that the maximum effectiveness of root hair enhancement in the lines tested would be wild type > *wer myb23* > *cpc try* (*M*_1_/*M*_2_ = 139, 134, 88, respectively). While cohesion enhancement in *wer myb23* and wild type were comparable, there was clearly a much lower value for *cpc try* plants (Table 2). These *M*_1_ and *M*_2_ parameter values reproduced the experimental observations with accuracy of 3%. These results further support the hypothesis that root hairs contribute to soil–root cohesion and soil erosion resistance. Our study provides evidence that root hairs enhance soil cohesion.

**Table 2:** Model parameters of the function describing the amount of reinforcement (i.e. additional cohesion) provided by root hairs in different *Arabidopsis thaliana* mutants.

	Wild type	*wer myb23*	*cpc try*
*M*_1_ range	3.60 × 10^−3^–7.30 × 10^−3^	2.67 × 10^−3^–3.50 × 10^−3^	3.27 × 10^−3^–4.36 × 10^−3^
*M*_1_*/M*_2_ range	138–142	116–141	80–97

*M*_1_ is the initial enhancement rate, *M*_2_ = *M*_1_/*M*_max_, which is the ratio of the initial enhancement rate to the maximum enhancement.

## Discussion

This study is the first to our knowledge to show that root hairs improve the erosion reduction potential of plant roots. We show that root hairs on Arabidopsis plants contribute to increased plant attachment to a gel medium, uprooting resistance from soil and compost, and reduce water erosion rates to almost zero in our experimental system compared to hairless roots, which showed no such effect even at high planting densities. While the lack of root hairs consistently reduced plant–soil cohesion compared to wild-type roots, root hair overproduction helped roots resist uprooting from both sterile gel and clay soil growth media more than wild type or performed similarly to wild type in compost (Figs. 2c and 3d). Moreover, *wer myb23* and wild-type roots reduced erosion at similar rates compared to bare soil (Fig. 4c, d). These variations in the measured effects of root hairs on soil erosion between genotypes with and without root hairs suggest that there are additional components of the root–soil interface that contribute to or have limited effects on soil cohesion.

There are contradictory reports in the literature of whether root hairs do or do not assist in substrate adhesion or soil penetration in different plant species^26–29^. However, by using Arabidopsis mutants specific for single root traits, we were able to determine the relative contributions the presence of root hairs make to root-substrate cohesion without confounding species-specific contributions. Further research will be required to characterise how aspects such as plant species and age, soil type, total root hair surface area, root hair density and root hair length specifically affect plant–substrate interactions.

A predominant view in the literature is that plant carbon (C) is converted by soil microorganisms into compounds that increase soil cohesion^30^ and that soil structure is important for soil C storage^31^. While mycorrhizal fungi release glomalin-related soil proteins and other exopolymers that affect soil aggregate stability^32^, Arabidopsis is not known to form mycorrhizal associations; however, there are evidence that root hairs can alter soil pore space and connectivity between these pores in the rhizosphere^11^. Indeed, we found that root hairs increase the adhesive strength of seedlings in a sterile root–gel system in the absence of microorganisms (Fig. 2), suggesting that root hairs alone account for substrate-adhesive properties.

Future work will explore the physical and biochemical aspects unique to root hairs that contribute to their soil–root binding abilities. In this respect, it is interesting to note that Akhtar et al.^33^ used a novel assay to identify polysaccharides important for soil cohesion, including chitosan, β-1,3-glucan, gum tragacanth, xanthan and xyloglucan. Similarly, Galloway et al.^15^ found that xyloglucan, a component secreted by a wide range of angiosperm roots, can increase soil particle aggregation. Building on these recent studies and our current results, we postulate three potential mechanisms by which plant root hairs might reinforce soil: (i) substrate components such as gel molecules or soil aggregates bind directly to root hair surfaces; (ii) root hairs release exudates that reinforce soil; and (iii) root hairs release exudates that are processed into material that reinforces soil by microbes. The approaches applied in this study can quantify relative contributions of other root traits to soil cohesion/erosion. The application of our findings and experimental approach will inform the selection or modification of root properties that could reduce soil erosion in agricultural, recreational and civil engineering contexts. Understanding how root hairs specifically affect plant–soil interactions improves our investigation of plant biology and has applications in soil and land preservation and maintenance under changing climate conditions.

## Methods

### Plants, soil and growth conditions

All *Arabidopsis thaliana* (L). Heynh mutants were in the Columbia (Col-0) background except *cpc try*, which was produced from a Col-0 × Wassilewskija cross and repeatedly backcrossed to Col-0. Plants were grown in controlled environment rooms at 20–22 °C, 60% humidity and 16 h light cycle (light intensity, 120–145 μmol m^−2^ s^−1^). Plants for the centrifugal root–gel attachment assay were sown onto the surface of gel medium. Plants for uprooting assays were grown on sieved (7 mm), clay soil (42.1% clay, 38% silt, 19.9% sand), or a sieved (7 mm) compost/sand mix (3:1 Levington UK F2 compost and J Arthur Bowers UK horticultural silver sand). Plants for erosion assays were grown on sieved (7 mm) clay–loam soil (27% clay, 36% silt, 37% sand). Gravimetric soil moisture content prior to the erosion tests ranged from 26% to 29%. Soil composition was determined using a sedigraph with a hexametaphosphate pre-treatment and ultrasonic bath. The United States Department of Agriculture standard was used to define soil textural description^34^. All soil was frozen at −50 °C to limit microbes and insects before use.

### Centrifugal gel-adhesion assay

Seeds were surface sterilised in 10% bleach, 0.05% Triton X-100 for 15 min, washed five times with sterile water and stratified at 4 °C in the dark for 48 h^35^. Ten sterile seeds were sown in two horizontal rows onto 90 mm Petri plates (Thermo Scientific RC2260) containing 1/2 Murashige and Skoog basal medium (Sigma M5519) with 1% (w/v) sucrose and 1% (w/v) agar (Sigma A1296), pH 5.7 and grown vertically for 5 days. Seedlings were spaced 1 cm apart and any seedlings touching each other were excluded during experimental reporting. Plates were placed inverted into a hanging basket centrifuge (Beckman Coulter Allegra X-30R Centrifuge) and subjected to 1-min incremental increases in centrifugal force of 720, 1018, 1247, 1440 and 1611 RPM (100, 200, 300, 400 and 500 × *g*). The proportion of seedlings that detached from the gel surface was determined between each speed. Data were collected for 87 wild type (Col-0), 87 *wer myb23*, 91 *35S::RSL4*, 94 *rsl4* and 88 *cpc try* seedlings. We report the results of a single experiment, which are representative of at least two independent experiments.

### Calculations and statistics for the gel adhesion assay

We determined the perpendicular plane of rotation of the hanging buckets within the enclosed centrifuge mathematically. The bucket is attached 70 mm from the axis of rotation, which also corresponds approximately to the surface of the gel in the plate and is free to swing about the axis. The centre of the bucket and plate mass lies on the axis of the bucket at a distance *l* from its attachment point. We assigned *m* as the mass of the bucket and plate, *ω* as the angular velocity (in radians per second) and *θ* as the inclination of the bucket to the vertical. The centrifugal force acting on the bucket was at least 0.07*mω*^2^ Newton, and the gravitational force is *mg* Newton. To define the balancing moments about the attachment point:

1 $$0.07\,m\omega ^2l\,\sin {\uptheta} \, < \, mgl\,\cos \theta.$$

Thus, $\tan \theta \, < \, g/(0.07\omega ^2)$. From the centrifuge documentation, *ω* is 720 *√n* rpm where *n* (i.e. the speed setting) is 1, 2, …, 9. We calculated that *θ* at the slowest rotation setting is less than 1.41° and, therefore, assumed that the bucket quickly swings out during centrifugation so that the Petri plates are orientated perpendicular to the plane of rotation. Hence, the seedlings experience a centrifugal force that can peel them away from the gel.

The maximum force resisted by the seedlings was used as a measure of root–gel adhesion. The angular velocity (*ω*) and diameter of the centrifuge (i.e. the distance between the seedling and the axis of rotation =70 mm and the aerial tissue weight of each seedling were used to calculate the maximal centrifugal force (Fc (kg m  s^−2^)) that was experienced by each seedling (mass, *Ms* (kg)) at each centrifugal speed:

2 $${\mathrm{Fc}} = Ms \times {\mathrm{radius}} \times \omega ^2.$$

We applied a Cox hazard function regression model^22^ to statistically test for differences between the risk of detachment for each root hair mutant relative to wild type. We set up a priori contrasts and used the coxph function with exact treatment of ties within the survival package in R^36^. We censored seedlings that remained attached to the gel medium after the maximum centrifugal speed setting because we did not determine what speed these seedlings would have detached from the gel.

For each regression model run, we report *P* value of the Wald test (*z*) and the hazard ratio with the upper and lower bound confidence intervals. Since the hazard ratio is an exponential coefficient that compares the risk of seedling detachment between root hair lines relative to wild type plants, the hazard ratio has been used as a measure of effect size^37^. Wild-type plants have a hazard ratio of one; root hair lines with a higher risk of detachment will have a hazard ratio above one, while lines with a lower risk of detachment will have a hazard ratio below one.

### Plant uprooting from soil and compost

Individual plants from multiple transgenic lines were grown and uprooted at the same time as control for run effects. Single, centrally placed plants were grown in 375 cm^3^ pots containing the same amount of soil. A polytetrafluoroethylene-coated (hydrophobic Tectane) aluminium washer was placed over the seedling within 3–4 days of germination. The plants were grown through the centre of the washer for 3–4 weeks until the plants had mature rosettes but were not yet reproductive. Therefore, the rosette anchored the washer around the plant so that the cables of the tensile machine could be attached to the washer for uprooting force measurement. Pots were saturated overnight in 3 cm water and plants were uprooted from either a compost–sand mixture (*n* = 13 wild type, *n* = 16 *wer myb23*, *n* = 13 *cpc try*) or a clay soil (*n* = 17 all lines). Plants were pulled vertically from the soil using a tensile testing machine (Instron 3343 with a 10 Newton load cell 2519-201) at a constant speed of 5 mm min^−1^ (refs. ^38–41^). Force traces were analysed to obtain total energy expended (area under the curve), peak force (maximum force reached) and the magnitude of the incremental force drops. The Instron 10 Newton load cell is accurate to 0.25% from 0.05 kg m s^−2^ and the mean of force above this threshold was calculated. The significance of the difference was also tested with limits at 0.1 and 0.035 kg m s^−2^, which satisfied *P*  < 0.05.

After uprooting, plant material was recovered and RLD (km m^−3^) recorded. RLD is a root trait frequently used to estimate the erosion-reducing potential of plant species and select the most suitable species for controlling soil erosion processes^42–45^. To determine RLD, the soil and root complex was washed thoroughly over a 0.7 mm sieve before manually separating the roots from the soil. The dry weights of these roots were used to determine plant RLDs by converting the root masses into root lengths using specific root length (i.e. root length per unit mass) values for each genotype. To obtain root specific length values, at least 10 m of root per genotype from at least three representative plants were separated into single strands on a high contrast background, photographed, measured in ImageJ^46^, dried and weighed. The root specific length values (m mg^−1^ root ± 1 standard deviation) for wild type, *cpc try* and *wer myb23* were 0.63 ± 0.04, 0.43 ± 0.07 and 1.02 ± 0.31 m mg^−1^ in clay soil and 0.51 ± 0.03, 0.39 ± 0.02 and 0.53 ± 0.02 m mg^−1^ in compost, respectively.

Uprooting data were analysed with a linear modelling framework that used the lm() function in ‘R’ (3.0.3) to investigate the variable-under-investigation/RLD relationship and how it is affected by the mutant background (Supplementary Table 1). Residuals were normal.

### Calculations and statistics for plant uprooting

Since the root–soil system responds to the uprooting force by a combination of deformation and damage, the peak force and total energy expended are macroscopic measures of root–soil cohesion. Let *f* (kg m s^−2^) be the uprooting force corresponding to a deformation *x* (m). Allowing for damage we may write generally that:

3 $$f\left( x \right) = k\left( x \right)x$$

for *0* < *x* < *x*_*p*_, where the function *k*(*x*) > 0 is a macroscopic elastic modulus and *x*_*p*_ is the deformation corresponding to the peak force.

At the peak force, $f( {x_p}) = k(x_p)x_p$, the system sustained critical damage and force decreases with subsequent deformation:

4 $$f\left( x \right) = k(x_p)x_p + h(x - x_p)(x - x_p)$$

for *x*_*p*_ < *x* < *x*_*u*_ where *h(x)* < *0* and *x*_*u*_ is the deformation corresponding to uprooting.

At uprooting, the force decreases to 0 and

5 $$k( {x_p} )x_p + h( {x_u - x_p} )( {x_u - x_p} ) = 0$$

and the total energy expended in uprooting is given by the integral *f* from 0 to *x*_*u*_.

The (possibly non-differentiable) functions *k*(*x*) and *h*(*x*) can vary from plant to plant and determine the mechanical properties of the system. We chose the peak force *f*(*x*_*p*_) and total energy expended, *E* (kg m^2^ s^−2^), as a measurement of the mechanical resistance as they are both functions of *k* and *h* and allow for the comparison of the mechanical resistance of the different mutants.

From our measurements, we found statistically significant differences between the mutants for *f*(*x*_*p*_) and *E*, which imply statistically significant differences for the functions *k* and *h* between mutants.

### Root reinforced soil resistance against concentrated flow erosion

Plants were grown in sieved clay–loam soil (dry soil bulk density 1.07–1.27 g cm^−3^) in boxes with inner dimensions 250 × 250 × 150 mm fitted with a weed suppression mat. Different plant densities of 9, 16, 32, 49, 81 and 100 plants in each box box (i.e. a plant density range of 144–1600 plants m^−2^) were established, which corresponded to shoot densities between 0.15 and 2.37, 0.37 and 1.72, and 0.57 and 2.66 kg m^−2^ for wild type, *cpc try* and *wer myb23*, respectively. All boxes were tested for erosion resistance at the same developmental stage after about 5 weeks growth (i.e. shortly after bolting). Data were collected from 18 boxes with wild-type roots, 17 with *cpc try* roots or 27 with *wer myb23* roots.

Immediately prior to erosion, the boxes were saturated by capillary rise, photographed and the aerial tissue and weed mats were removed. Gravimetric soil moisture content before erosion tests was between 0.26 and 0.29 g g^−1^. Erosion assays were conducted in a water flume with a 28° slope similar to that used in previous studies^47,48^. The soil surface was exposed to 1 l s^−1^ of running water at corresponding mean bottom flow shear stresses between 13 and 24 Pa. The run-off water and eroded soil was captured for 5 s at 10 s intervals for 2 min. Flow velocity was measured using the dye tracing technique^49^. A bare soil control experiment was prepared at the same time and in the same way as the planted samples. Bare soil boxes were placed in growth rooms for the same period of time with a weed control sheet on the surface to prevent algae and moss growth and watered along with the planted boxes. Sediment concentration was used to calculate sediment detachment rates (kg m^2^ s^−1^) for each collection interval. Soil detachment rates were averaged out per sample and normalised using the extrapolated soil detachment value for a root density of zero.

Immediately after each experimental run, roots were separated from the soil by hand washing using an adapted version of the method previously described^50^. The recovered roots were washed and weighed so that RLD (km m^−3^) could be calculated using specific root length (root length/unit mass) measured from 10 wild type, *cpc try* and *wer myb23* root samples, which were 0.44 ± 0.05, 0.52 ± 0.10 and 0.58 ± 0.15 m mg^−1^, respectively (Fig. 4d).

### Calculations and statistics for resistance to erosion

Nonlinear regression models with functional forms that corresponded to exponential decay to a constant value were fitted through the experimental data describing the erosion-reducing potential of root-permeated soils as a function of the root variable RLD. In order to calculate the error on the modelled curves due to parameter uncertainty, Monte Carlo simulations were performed by perturbing the parameter estimates 10,000 times from a set of parameter values randomly chosen from a normal probability distribution with mean and standard deviation equal to the estimated value and its standard error, respectively. Hence, the uncertainty bounds on the modelled curves indicate the 95% confidence interval of the fitted functions. Where the modelled curves do not fall within another curve’s uncertainty bound, they are significantly different at *P* <  0.05.

### Derivation of root reinforcement

Root reinforcement was defined as the difference between bare soil cohesion and the cohesion of soil containing roots. Soil cohesion values for bare and root-containing soils were established by back calculation of transport capacity efficiencies and corresponding soil cohesion values. Measured soil detachment rates were set equal to modelled soil detachment rates using the EUROSEM^51^ equation for modelling detachment by runoff. Therefore, the only unknown parameter is the flow detachment efficiency coefficient, *β*, derived from the measured soil detachment rate (ASD, g cm^−2^ s^−1^), flow and sediment properties:

6 $$\beta = \frac{{\mathrm{{ASD}}}}{{\root {{B_{\mathrm{D}}}} \of {{\frac{{4d_{50}\left( {\rho _{\mathrm{s}}\, -\, \rho _{\mathrm{w}}} \right)g}}{{3C_{\mathrm{D}}\rho _{\mathrm{W}}}}}}C_{\mathrm{{TC}}}}},$$

where *ρ*_s_ is density of the detached sediments (g cm^−3^) where 2.65 g cm^−3^ was used^52^, *ρ*_w_ (g cm^−3^) is density of water, *g* is gravity acceleration, *d*_50_ (μm) is median grain-size diameter (equalling 16 ± 1.14 μm for our clay–loam soil), and *C*_D_ the drag coefficient calculated from a formula using the grain Reynolds number that is calculated from the flow characteristics of the experimental runs and from the average grain size of the soil.

The value of soil cohesion that corresponds to this *β* value was calculated using the following empirically derived equation^53,54^:

7 $$C = ( - 1/0.85)\ln (\beta /0.79).$$

The full method for back calculation of corresponding soil cohesion when soil detachment and flow characteristics were measured was as described^7^.

### Mechanistic modelling

For each mutant, the enhancement of soil cohesion with increasing RLD was quantified. We first determined the volume occupied by the Arabidopsis root system. From the flow rate and velocity, we deduced the shear force acting on the soil surface. This force is resisted by the cohesion of root-reinforced soil, but not in a homogeneous manner because resistance is strongest along the primary root, decreases radially outwards, and depends on depth; we use knowledge of root architecture to model this (Fig. 4a). Erosion occurs at regions where the shear force exceeds the local soil cohesion. The model integrates fluid flow, root architecture, soil mechanics and debris entrainment as described below and in the literature^16,55–57^. The erosion depth increases over the course of the experiment and reaches a maximum value *R*.

### Fluid flow

The water volume flow rate (*Q*, m^3^ s^−1^) and surface velocity (V, m s^−1^) were experimentally measured. Since flow profile is assumed parabolic, the shear stress acting on the soil surface can be calculated as

8 $$\tau = (3gQd_{50}^2\sin \left( {28^\circ } \right))/(2VkW),$$

where *W* = 360 mm is the width of the flume and *k* is the bare soil permeability, for which we used the representative value 0.2273 μm^2^ ^58^.

### Soil-volume occupied by roots

The typical volume occupied by an Arabidopsis root system after 5 weeks of growth and the arrangement of the volume occupied by roots is described by a kite shaped structure with *W, D* and *d* as parameters describing the size of the root system (Fig. 4a). *D* and *W* describe root volume in horizontal and vertical directions, respectively, and *d* indicates the depth at which the root system has maximum lateral spread. For our experimental design, *D* is 100 mm, *d* is 20–30 mm and *W* is 100 mm. *R* and *r* are erosion parameters. The maximum erosion depth is given by *R* and the depth of the root system within *R* of the surface diagonally between plant stems is *r*. Hence, *r* is no larger than *R*. Here *R* = 50 mm and *r* varies from 23 to 42 mm depending on the number of plants. *r* was derived using eroded mass, bulk density and box dimensions. *R* was a set value. Each experiment was stopped when erosion depth reached 50 mm^59^.

### Soil mechanics

Soil is modelled as an isotropic nonlinear elastic material which has limiting behaviour that tends to that of a material described by Mohr–Coulomb theory as a brittle material. We call this an isotropic ‘elastic-Coulomb' material that is eroded when the shear stress (*τ*) reaches a critical value determined by the Coulomb criterion

9 $$\tau = - \mu N + c,$$

where *µ* is a friction coefficient, *c* is the cohesion of soil containing plant roots and *N* is the normal stress. The cohesion of soil containing plant roots depends on RLD. The maximum cohesion, *c*_Max_, that occurs at the tap root is

10a $$c_{{\mathrm{Max}}} = c_{{\mathrm{Bare}}}(1 + \gamma \left( {RLD_{\rm{T}}} \right)R),$$

where *c*_Bare_ is the cohesion of bare soil and *γ*(*RLD*T), in mm^−1^, is the increased depth-integrated soil cohesion due to roots, which is a function of RLD_T_ as the true root length density, or the total root length divided by the volume of the regions occupied by the root. Similarly, the minimum soil cohesion occurs furthermost from the tap root and is

10b $$c_{{\mathrm{Min}}} = c_{{\mathrm{Bare}}}(1 + \gamma \left( {{\mathrm{{RLD}}}_{\rm{T}}} \right)r).$$

In Eq. (10) *r* and *R* represent the erosion parameters defined in the previous paragraph. The function *γ(x)* has two properties that are approximately linear for small values of *x* and saturates to a constant value at large *x* (i.e., there is a limit to the enhancement root hairs have on soil cohesion). We chose a simple function that has these properties:

11 $$\gamma \left( x \right) = M_{\mathrm{{max}}}\tan {\mathrm{h}}(M_1x/M_{\mathrm{{max}}}).$$

The maximum amount of root hair enhancement is given by *M*_max_, since tan h takes values no larger than 1. For very low root length densities *x*, $\gamma (x) \approx M_1x$, hence the initial slope (i.e. the rate of enhancement at low RLD_T_ is given by *M*_*1*_ because tan h (*x*) ≈ *x* for small *x*. For analysis is it useful to define the new parameter, *M*_2_ =  *M*_1_/*M*_max_, which is the ratio of the initial enhancement rate to the maximum enhancement. *M*_1_, *M*_2_ are thus parameters describing the reinforcement plant roots provide and depend on the micro-scale properties of plant roots. To model the regular periodic array of plants, the cohesion *c* in Eq. (9) is allowed to spatially vary in a sinusoidal manner taking values between *c*_Min_ and *c*_Max_. Therefore, we obtain a mechanical model for erosion as a function of RLD_T_ (Fig. 4c, d). Since we have controlled for the root architecture (factor RLD_T_ in Eq. (10) and function *γ* in Eq. (11)), *M*_1_ and *M*_2_ quantify the amount of cohesion enhancement by micro-scale root traits and allow us to compare the effectiveness of different mutants in controlling erosion.

### Analysis of root morphology for root phenotyping

Surface sterilised seeds were stratified at 4 °C for 48 h then grown on square 12 cm^2^ plates containing nutrient medium^60^ with 1% sucrose 1% Phytagel (Sigma Aldrich), pH 5.7, and sealed with Parafilm (Bemis, NA). Plates were incubated vertically for 10–11 days, when the root tips of wild-type plants reached within 1 cm of the bottom of the plate. For each genotype, 20 single seeded plates and 5 plates with 5 seeds were used to measure and compare the growth of single and grouped plants. Each set was compared with a wild-type control. ‘Root depth’ was the vertical distance the root tip had progressed down the plate. Root-hair counts were taken using dark field lighting on a Leica MZ FLIII microscope. A Nikon D50 camera with a polarising filter and SPOT image capture software (SPOTIMAGING) was used to capture microscope images. Image analysis was conducted with a combination of ImageJ^42^ and RootNav software^61^. Plants for X-raying were grown in 200 μl pipette tips filled with sieved clay soil for 5–7 days. Tips were scanned with a Nikon XT H 225 ST CT scanner (settings: energy: 90 kV, current 60 (μA) exposure 1 s, 5 frames averaged per projection, voxel size = 0.00278056).

### Statistics and reproducibility

For all experiments, biological replicates for each root hair line were randomly selected from pools of seed containing genetically identical individuals for the trait of interest.

### Centrifugal gel-adhesion assay

Ten seedlings of each line were sown onto a single Petri plate containing 30 ml gel medium. For an individual experiment, there was a replicate size of over *n* *=* 70 for each line. To account for potential heterogeneity in gel thickness and composition between Petri plates, angular rotation that each seedling experienced and spin number were incorporated as covariates in our analysis. The Petri plates were oriented vertically at approximately 80°, in stacks of five in a controlled growth room using a Latin Square design. Statistical analysis was performed in R with a Cox hazard function regression that included all covariates listed above in our statistical model and were removed only if they had no significant effect. The reported effect size is the hazard ratio, which includes lower and upper bound confidence intervals. *P* values were calculated from the Wald Statistic (*z*) with a significance level of 0.05. This study was conducted blind. The results for each line presented in this paper are representative of at least two independent experiments, although we have observed similar results in at least five independent trials, each run by different lab members.

### Plant uprooting from soil and compost

Pots containing single plants were grown in trays containing six pots, organised in a Latin square design in a temperature and light-controlled growth room, and were rotated every 2 days to prevent edge effects. The tensile testing machine used to uproot plants was tested prior to conducting an experiment. The night before an experiment, pots were placed in 3 cm water to allow saturation to ensure a consistent soil moisture level. Uprooting experiments were conducted blind to genotype. Between 13 and 17 individual plants were uprooted for each genotype. Pairwise comparisons of peak force, work done and force drop magnitude were conducted on wild-type plants relative to root hair mutants using the lm() function in *R*. The number of samples tested per line was sufficient for linear regression and pairwise comparisons of regression parameters for all genotypes. A significance level of 0.05 was used for all regression parameters.

### Root reinforced soil resistance against concentrated flow erosion

We grew 9, 16, 32, 49, 81 or 100 plants per box in a sieved clay-loam soil medium. For each experimental replicate, plants from all three lines were grown simultaneously in a controlled environment growth room to keep growing conditions as homogeneous as possible. On each experimental day at least five boxes were tested from at least two different lines. Different planting densities were used to obtain variation in root density. In total, 18 (wild type), 17 (*cpc try*) and 27 (*wer myb23*) soil boxes were tested. The number of soil boxes tested per line was sufficient to conduct nonlinear regression models and the comparison of regression parameters for different lines. Statistical analysis was performed in IBM SPSS Statistics 25 using the nonlinear regression function and in MATLAB R2014a (MathWorks, Natick, Massachusetts, USA) for computing the error bounds on the modelled regressions. To compute the regression error bounds, we perturbed the parameter estimates 10,000 times from a set of parameter values randomly chosen from a normal probability distribution with mean and standard deviation equal to the estimated value and its standard error, respectively. Therefore, when the modelled curves do not fall within another curve’s uncertainty bound, this indicates a significant difference between lines at *P* < 0.05.

### Reporting summary

Further information on research design is available in the Nature Research Reporting Summary linked to this article.

## Supplementary information

Supplementary Movie 1

Supplementary Information

Reporting Summary

Description of Additional Supplementary Files

Peer Review File

## Data Availability

All figures have associated raw data. The data that support the findings of this study are available from the University of Bristol’s research data repository, data.bris, at 10.5523/bris.1vca1omqff8bj2a7rpkbcgxc7y. Data collection and data analysis codes are available upon request from the authors. There are no restrictions on data availability.

## Code availability

The following codes are available from the authors upon request: To detect vertical force drops during uprooting in Python 2.7.9 (Python Software Foundation), to analyse uprooting data using ‘R’ 3.0.3 (R Foundation), for Monte Carlo analysis and the mechanistic erosion model in MATLAB R2014a (MathWorks, Natick, Massachusetts, USA).
