# Supplementary material for: Micro-scale interactions between Arabidopsis root hairs and soil particles influence soil erosion
Source: Commun Biol. 2020 Apr 3;3:164. doi: 10.1038/s42003-020-0886-4 (PMC7125084; doi:10.1038/s42003-020-0886-4)
Supplement: Supplementary file 2 — Supplementary Information [file 42003_2020_886_MOESM2_ESM.pdf]

Supplementary Table 1. Linear model results and pairwise comparison for the uprooting experiment in soil and compost. All results are in comparison to wild type (Col-0). Dashes indicate no significant difference. Analysis was conducted in 'R' 3.0.3 using lm().

Substrate	Variable	Significance of interaction	Significance of line effect at mean root length density	Line	Pair-wise comparison to Col-0<br>(t= Intercept of the mean root length density)<br>(± standard deviation of residuals)	Pairwise comparison to Col-0<br>(t= Parameter of interaction with root length density)<br>(± standard deviation of residuals)
**clay soil**	Peak Uprooting Force (N)	$F_{2,54} = 13.27$<br>$P = 2.21x10^{-5}$	$F_{2,56} = 57$<br>$P = 4.959x10^{-14}$	Wild type	(±0.33)	(±0.33)
				cpc try	t =-6.745<br>$P = 1.07x10^{-8}$<br>d.f = 56<br>(±0.26)	t = -3.034<br>P = 0.00376<br>d.f = 54<br>(±0.13)
				wer myb	t =3.826<br>P = 0.00034<br>d.f = 56<br>(±0.41)	t = 2.605<br>P = 0.01194<br>d.f = 54<br>(±0.32)
	Work Done (mJ)	$F_{2,54} = 18.4$<br>$P = 8.937^{-7}$	$F_{2,56} = 30.44$<br>$P = 1.4x10^{-9}$	Wild type	(±6.54)	(±6.52)
				cpc try	t = -4.371<br>P = 0.000057<br>d.f. = 56<br>(±6.6)	t = -2.814<br>P = 0.006896<br>d.f = 54<br>(±1.8)
				wer myb	t = 3.44<br>P = 0.0011<br>d.f. = 56<br>(±13.3)	t = 3.807<br>P = 0.000372<br>d.f = 54<br>(±10.4)
	Mean Magnitude of Force Drop (N)	-	$F_{2,56} = 12.57$<br>$P = 3.293x10^{-5}$	Wild type	(±0.02)	-
				cpc try	t = -4.3<br>$P= 7.19x10^{-5}$<br>d.f = 56<br>(±0.015)	-
				wer myb	-	-
**compost**	Peak Uprooting Force (N)	$F_{2,41} = 4.15$<br>P = 0.02308	$F_{2,43} = 13.58$<br>$P = 2.98x10^{-5}$	Wild type	(±0.46)	(±0.42)
				cpc try	t = -4.953<br>$P = 1.30x10^{-5}$<br>d.f. = 43<br>(±0.38)	t = -2.394<br>P = 0.0216<br>d.f = 41<br>(±0.28)
				wer myb	-	-
	Work Done (mJ)	$F_{2,41} = 7.439$<br>P = 0.00183	$F_{2,43} = 12.3$<br>$P = 6.508x10^{-5}$	Wild type	(±14)	(±11.2)
				cpc try	t = -4.882<br>$P = 1.64x10^{-5}$<br>d.f. = 43<br>(±12.2)	t = -3.618<br>P = 0.000842<br>d.f = 41<br>(±8.82)
				wer myb	-	-
	Mean Magnitude of Force Drop (N)	-	-	Wild type	-	-
				cpc try	-	-
				wer myb	-	-
