# Supplementary material for: Micro-scale interactions between Arabidopsis root hairs and soil particles influence soil erosion
Source: Commun Biol. 2020 Apr 3;3:164. doi: 10.1038/s42003-020-0886-4 (PMC7125084; doi:10.1038/s42003-020-0886-4)
Supplement: Supplementary file 3 — Reporting Summary [file 42003_2020_886_MOESM3_ESM.pdf]

nature research

Corresponding author(s): COMMSBIO-19-1961-T, Claire Grierson

Last updated by author(s): 27.02.2020

Reporting Summary

Nature Research wishes to improve the reproducibility of the work that we publish. This form provides structure for consistency and transparency in reporting. For further information on Nature Research policies, see Authors & Referees and the Editorial Policy Checklist.

Statistics

For all statistical analyses, confirm that the following items are present in the figure legend, table legend, main text, or Methods section.

n/a	Confirmed	
☐	☒	The exact sample size (*n*) for each experimental group/condition, given as a discrete number and unit of measurement
☐	☒	A statement on whether measurements were taken from distinct samples or whether the same sample was measured repeatedly
☐	☒	The statistical test(s) used AND whether they are one- or two-sided<br>*Only common tests should be described solely by name; describe more complex techniques in the Methods section.*
☒	☐	A description of all covariates tested
☐	☒	A description of any assumptions or corrections, such as tests of normality and adjustment for multiple comparisons
☐	☒	A full description of the statistical parameters including central tendency (e.g. means) or other basic estimates (e.g. regression coefficient) AND variation (e.g. standard deviation) or associated estimates of uncertainty (e.g. confidence intervals)
☐	☒	For null hypothesis testing, the test statistic (e.g. *F*, *t*, *r*) with confidence intervals, effect sizes, degrees of freedom and *P* value noted<br>*Give P values as exact values whenever suitable.*
☒	☐	For Bayesian analysis, information on the choice of priors and Markov chain Monte Carlo settings
☒	☐	For hierarchical and complex designs, identification of the appropriate level for tests and full reporting of outcomes
☒	☐	Estimates of effect sizes (e.g. Cohen's *d*, Pearson's *r*), indicating how they were calculated

Our web collection on statistics for biologists contains articles on many of the points above.

Software and code

Policy information about availability of computer code

Data collection	For experiment 1, 2 and 3 data was manually inputted into excel. For experiment 2 (TD) to detect vertical force drops during uprooting, a code written in Python 2.7.9 (Python Software Foundation) is available from the authors upon request.
Data analysis	For experiment 1 (BE) RStudio version 1.1.463 was used running the coxph function (with 'exact ties') within the 'survival' package. For experiment 2 (TD) to analyse the uprooting data, a code written in 'R' 3.0.3 (R Foundation) is available from the authors upon request. For experiment 3, the code for the Monte Carlo analysis (JM) and for the mechanistic erosion model (BH), written in in MATLAB R2014a (MathWorks, Natick, Massachusetts, USA) is available from the authors upon request.

For manuscripts utilizing custom algorithms or software that are central to the research but not yet described in published literature, software must be made available to editors/reviewers. We strongly encourage code deposition in a community repository (e.g. GitHub). See the Nature Research guidelines for submitting code & software for further information.

Data

Policy information about availability of data

All manuscripts must include a data availability statement. This statement should provide the following information, where applicable:

- Accession codes, unique identifiers, or web links for publicly available datasets
- A list of figures that have associated raw data
- A description of any restrictions on data availability

Data management plan:
The following codes are available from the authors upon request: to run the coxph function (with 'exact ties') with the survival package in R 3.0.3, to detect vertical force drops during uprooting in Python 2.7.9 (Python Software Foundation), to analyse uprooting data using 'R' 3.0.3 (R Foundation), for Monte Carlo analysis and the mechanistic erosion model in MATLAB R2014a (MathWorks, Natick, Massachusetts, USA).
Fig 1, 2 and 3 have associated raw data. The raw data for these figures will be accessible and published on Cranfield University's data repository system (CORD, https://cord.cranfield.ac.uk/) before publication, and the paper will link to the underlying datasets. CORD is a public repository that does not issue datasets with

DOIs. There are no restrictions on data availability.

Field-specific reporting

Please select the one below that is the best fit for your research. If you are not sure, read the appropriate sections before making your selection.

☐ Life sciences ☐ Behavioural & social sciences ☒ Ecological, evolutionary & environmental sciences

For a reference copy of the document with all sections, see nature.com/documents/nr-reporting-summary-flat.pdf

Ecological, evolutionary & environmental sciences study design

All studies must disclose on these points even when the disclosure is negative.

Study description	Experiment 1 (BE): To test the differences between the root-gel adhesion properties of Arabidopsis lines with differing root hair properties (long root hairs, increased root hair density, short root hairs and no root hairs) relative to a wild type control line (Col-0). Plants were subject to incremental increases in centrifugal force (5 centrifugal speed settings were used) for one minute at a time. Detachment of plants from the gel medium were recorded at discrete time points, following a pulse of centrifugal force. A cox PH regression survival analysis was conducted on the data and the effect size reported is the hazard ratio (with the upper and lower bound confidence intervals). For each line, the replicate number was over 70n.<br>Experiment 2 (TD): Individuals from three Arabidopsis thaliana lines were grown and uprooted after they had grown for 3-4 weeks. Soil moisture content in the pots was equilibrated by placement overnight in 3 cm water. Rosette stage, non-reproductive plants were uprooted from either a compost-sand mixture (n=13 for wild type, n=16 for wer myb, n=13 for cpc try) or a clay soil (n=17 for all lines). Plants were pulled vertically from the soil using a tensile testing machine (Instron 3343 with a 10N load cell 2519-201) at a constant speed of 5 mm/min.<br>Experiment 3 (SDB): Soil detachment rates were measured on soil samples planted with 3 Arabidopsis thaliana lines at different planting densities. All samples were packed and irrigated equally before the test and grown for 4-6 weeks. Before the erosion test, the aboveground biomass was cut off so only the effects of the roots on the resistance of the soil to water erosion was accounted for. A hydraulic flume was used to test the erosion response and the detachment rates of the planted samples were expressed relative compared to the detachment rate measured on unvegetated samples that has been placed and irrigated in the controlled growth environment for the same amount of time. Root length density data was collected from the samples after the erosion experiments. n=18 soil boxes containing wild type roots, 17 (cpc try) and 27 (wer myb23). Non-linear regression analysis was then performed on the soil loss and root data, with Monte Carlo simulation used to plot error bounds on the modeled regressions.
Research sample	Experiment 1 (BE): five Arabidopsis thaliana lines were used with differing root hair properties including: Col-0 (wild type control line - normal root hair number and length); 35S::RSL4 (transgenic overexpressor line producing long root hairs); wer myb23 (mutant line producing an increased number of root hairs which are slightly longer); rsl4-1 (mutant line with a decrease in root hair number and length); and cpc try (mutant with no root hairs).<br>Experiment 2 (TD): three Arabidopsis thaliana lines were used with differing root hair properties including: Col-0 (wild type control line - normal root hair number and length); wer myb23 (mutant line producing an increased number of root hairs which are slightly longer) and cpc try (mutant with no root hairs).<br>Experminent 3 (SDB): three Arabidopsis thaliana lines were used with differing root hair properties including: Col-0 (wild type control line - normal root hair number and length); wer myb23 (mutant line producing an increased number of root hairs which are slightly longer) and cpc try (mutant with no root hairs).
Sampling strategy	Experiment 1 (BE): The detachment of over 80 seedlings of a particular Arabidopsis thaliana line (explained above) from the gel were recorded. No statistical methods were used to predetermine sample size. When the assay was initially designed, a sample size of 30 - 40 n was used, however, this caused a large margin of error and gaps within the survival curves so BE compared the error margins by repeating the experiment with replicate sizes of ~50n, ~70n and ~100n. BE found that a replicate size of at least 70n is sufficient.<br>Experiment 2 (TD): 13-17 replicates for each Arabidopsis thaliana line were tested in the uprooting assay (see above for exact number of replicates per line). Plots of the total work done (area under curve), peak force and magnitude of force drops during the uprooting of wild type , hairless cpc try and hairy wer myb23 mutant plants from compost and a clay soil were made and pairwise comparison was performed in R using lm() on the regression parameters from the linear regressions predicting work done, peak force or magnitude of force drops in function of root length density, for the root hair deficit and root hair overproducing lines compared to the wild type line. This number of samples tested per line was found to be enough to perform linear regression on and to be able to do a pairwise comparison of regression parameters for the different lines.<br>Experiment 3 (SDB): 17-27 replicates for each Arabidopsis thaliana line were tested (see above for exact numbers) in the erosion assay. Several batches of plants from the three lines were grown in a controlled environment growth room so growing conditions were always the same. Samples affected by disease or with a poor germination were not taken forward to the erosion tests. 4-5 days after germination of the first seedlings, spare seedlings were transplanted into the spots were no seedlings had germinated to obtain the set planting densities of 9, 16, 32 and 81 plants per box. These different planting densities were established to obtain a variation in root densities. This number of samples tested per line was found to be enough to perform non-linear regression on and to be able to do a comparison of regression parameters for the different lines. The regression models fitted through the experimental erosion data were robust with relatively narrow 95% error bounds (simulated with Monte Carlo).
Data collection	Experiment 1 (BE): BE recorded the data into a lab book, this was then inputted into excel for analysis in R.<br>Experiment 2 (TD): TD recored the data into a lab book, this was then inputted into excel for analysis in R.<br>Experiment 3 (SDB/TD): SDB and TD recorded the data into a lab book, this data was then inputted into excel and analysed with SPSS and MATLAB. BH used the experimental data to calibrate the mechanistic soil cohesion model in MATLAB.
Timing and spatial scale	Experiment 1 (BE): n/a as plants were grown in controlled growth conditions. This assay is conducted when the plants are 5-6 days

Timing and spatial scale	old (as this is the appropriate age for them to fit on the gel medium within a petri plate and not cause the gel to shatter).<br>Experiment 2 (TD): n/a as plants were grown in controlled conditions. This assay is conducted when plants were 3-4 w old (rosette stage, non reproductive plants) and not pot bound yet 375 cm3 pots .<br>Experiment 3 (SDB): n/a as plants were grown in controlled conditions. This assay is conducted when plants were 4-6 weeks old (non reproductive plants, roots have fully explored 0.0086 m3 soil box, not pot bound yet).
Data exclusions	No data was excluded from non of the experiments.
Reproducibility	Experiment 1: BE optimized and enhanced the resolution of the centrifuge assay using the same lines that were previously used by KMS. When KMS conducted the assay (prior to method optimization) the root-gel adhesion properties of rsl4-1, cpctry, and 35S::RSL4 plants relative to wild type (Col-0) plants were comparable. However, no difference was found between wer myb23 and Col-0 plants. Following assay optimization by BE, three independent pairs of undergraduate level students conducted the assay using col-0, cpc try, 35S:RSL4 and wer myb23 plants (no rsl4-1 plants were used) rsl4-1 blind. Across all three pairs, the results were consistent and comparable to the results reported herein.<br>Experiment 2: The uprooting assay method was developed by AM and further optimized by TD. The measurements on the different lines were conducted blind, the results were consistent and comparable between different operators.<br>Experiment 3: SDB, TD, JM and LW conducted flume experiments. The flume experiment methods were developed by SDB and further optimised with help from TD, JM and LW. The sample coding was done blind. The results were consistent and comparable between different operators.
Randomization	Experiment 1 (BE): Replicates for particular line were randomly selected from pools of seed containing individuals that are genetically identical for the trait of interest (root hair characteristics). In this experiment, 10 seedlings of a particular line are sown onto a single petri plate containing 30 ml of gel medium (there were at least 8 seed-sown petri plates per line). To account for potential heterogeneity in gel thickness and composition between petri plates, plate number was included as a covariate. As the angular rotation that each seedling experienced is likely to be position-dependent, seedling position was incorporated as a covariate. Since only 4 plates could be spun in a centrifuge for a single run, spin number was incorporated as a covariate. These covariates were not reported in the final model as they had no significant interaction. When BE grew the plants, petri plates were vertically stacked in a controlled growth room. To account for potential positional environmental heterogeneity, BE used a Latin square design.<br>Experiment 2 (TD): Replicates for a particular line were randomly selected from pools of seed containing individuals that are genetically identical for the trait of interest (root hair characteristics). In this experiment, single, centrally placed plants were grown in 375 cm3 pots. All plants were grown in controlled growth conditions.<br>Experiment 3 (SDB): Replicates for a particular line were randomly selected from pools of seed containing individuals that are genetically identical for the trait of interest (root hair characteristics). In this experiment, 9, 16, 32 or 81 seeds were planted in each 0.0086 m3 soil box. 4 different batches of plants were grown. Two or three lines were grown simultaneous each time. All plants were grown in controlled growth conditions.
Blinding	Experiment 1 (BE): the study was conducted blind - BE got a colleague to re-label each sample prior to conducting the centrifuge assay. BE was informed of the lines identity when inputting the data in excel for statistical analysis.<br>Experiment 2 (TD): the experiment was conducted blind - coding was used for the samples and the sample identity was linked afterwards to the codes when the data were inputted into excel.<br>Experiment 3 (SDB): the experiment was conducted blind. After cutting off the above ground biomass, a code was give to each box and the samples were randomly tested across all lines.

Did the study involve field work? ☐ Yes ☒ No

Reporting for specific materials, systems and methods

We require information from authors about some types of materials, experimental systems and methods used in many studies. Here, indicate whether each material, system or method listed is relevant to your study. If you are not sure if a list item applies to your research, read the appropriate section before selecting a response.

Materials & experimental systems

n/a	Involved in the study
☒	☐ Antibodies
☒	☐ Eukaryotic cell lines
☒	☐ Palaeontology
☒	☐ Animals and other organisms
☒	☐ Human research participants
☒	☐ Clinical data

Methods

n/a	Involved in the study
☒	☐ ChIP-seq
☒	☐ Flow cytometry
☒	☐ MRI-based neuroimaging
