# Supplementary material for: Micro-scale interactions between Arabidopsis root hairs and soil particles influence soil erosion
Source: Commun Biol. 2020 Apr 3;3:164. doi: 10.1038/s42003-020-0886-4 (PMC7125084; doi:10.1038/s42003-020-0886-4)
Supplement: Supplementary file 4 — Description of Additional Supplementary Files [file 42003_2020_886_MOESM4_ESM.pdf]

## Description of Additional Supplementary Files

**File Name**: **Supplementary Movie 1**
**Description**: Soil erosion by concentrated flow is reduced by root hairs.
