# Supplementary material for: Micro-scale interactions between Arabidopsis root hairs and soil particles influence soil erosion
Source: Commun Biol. 2020 Apr 3;3:164. doi: 10.1038/s42003-020-0886-4 (PMC7125084; doi:10.1038/s42003-020-0886-4)
Supplement: Supplementary file 5 — Peer Review File [file 42003_2020_886_MOESM5_ESM.pdf]

Editorial Note: *This manuscript has been previously reviewed at another Nature Research journal. The manuscript was considered suitable for publication without further review at Communications Biology*
